# Angiotensin-(1-7) relieves behavioral defects and α-synuclein expression through NEAT1/miR-153-3p axis in Parkinson’s disease

**DOI:** 10.18632/aging.206028

**Published:** 2024-10-17

**Authors:** Qing Gao, Xiaoyuan Li, Ting Huang, Li Gao, Siyu Wang, Yang Deng, Feng Wang, Xue Xue, Rui Duan

**Affiliations:** 1 Department of Neurology, Nanjing First Hospital, Nanjing Medical University, Nanjing 210006, Jiangsu, P.R. China; 2 Department of Nuclear Medicine, Nanjing First Hospital, Nanjing Medical University, Nanjing 210006, Jiangsu, P.R. China; 3 Department of Neurology, Ren Ji Hospital, Shanghai Jiao Tong University School of Medicine, Shanghai 200127, P.R. China; 4 Department of Neurology, Nanjing First Hospital, China Pharmaceutical University, Nanjing 210006, Jiangsu, P.R. China

**Keywords:** Parkinson’s disease, angiotensin-(1-7), α-synuclein, lncRNA NEAT1, miR-153-3p

## Abstract

Parkinson’s disease (PD) is the second most common neurodegenerative disorder, whose characteristic pathology involves progressive deficiency of dopaminergic neurons and generation of Lewy bodies (LBs). Aggregated and misfolded α-synuclein (α-syn) is the major constituent of LBs. As the newly discovered pathway of renin-angiotensin system (RAS), Angiotensin-(1-7) (Ang-(1-7)) and receptor Mas have attracted increasing attentions for their correlation with PD, but underlying mechanisms remain not fully clear. Based on above, this study established PD models of mice and primary dopaminergic neurons with AAV-hα-syn(A53T), then discussed the effects of Ang-(1-7)/Mas on α-syn level and neuronal apoptosis for these models combined with downstream long non-coding RNA (lncRNA) and microRNA (miRNA). Results showed that Ang-(1-7) alleviated behavioral impairments, rescued dopaminergic neurons loss and lowered α-syn expression in substantia nigra of hα-syn(A53T) overexpressed PD mice. We also discovered that Ang-(1-7) decreased level of α-syn and apoptosis in the hα-syn(A53T) overexpressed dopaminergic neurons through lncRNA NEAT1/miR-153-3p axis. Moreover, miR-153-3p level in peripheral blood is found negatively correlated with that of α-syn. In conclusion, our work not only showed neuroprotective effect and underlying mechanisms for Ang-(1-7) on α-syn *in vivo* and *vitro*, but also brought new hope on miR-153-3p and NEAT1 for diagnosis and treatment in PD.

## INTRODUCTION

As the second most common neurodegenerative disorder, Parkinson’s disease (PD) seriously affects the quality of life, thus constituting a huge health encumbrance to patients, their families and the whole society [1]. The pathological hallmark of PD includes neural inclusions in the form of Lewy bodies (LBs) and lack of dopaminergic cells in the substantia nigra (SN) [2]. Considering that aggregated and misfolded α-synuclein (α-syn) is the major constituent of LBs, more and more attentions have been spent on the underlying mechanisms and therapeutic strategies targeting α-syn pathology [3].

The angiotensin-derived peptide Angiotensin-(1-7) (Ang-(1-7)) and its receptor Mas are newly discovered compositions of renin-angiotensin system (RAS) [4]. Different from classical Angiotensin II/AT1 receptor axis of RAS, Ang-(1-7)/Mas pathway is able to counteract cellular senescence and inflammation and exerts a protective effect [5]. Emerging evidence have suggested that Ang-(1-7)/Mas is closely associated with quite a few neurological problems especially neurodegenerative diseases [6]. Cao C reported that Ang-(1-7) infusion could improve cognitive deficit and skeletal muscle impairment in an AD mouse model [7]. Kangussu LM also showed that the imbalance of Ang-(1-7)/Mas and Angiotensin II/AT1 axis may act an indispensable role in Huntington’s disease [8]. As for PD, some clues have indicated Ang-(1-7) could alleviate rotenone-induced oxidative damage in CATH.a neurons [9]. However, the specific mechanisms of Ang-(1-7)/Mas in pathological process of α-syn for PD remain not fully understood.

Actually, more and more researches have focused on the biological implications of microRNAs (miRNAs) on α-syn pathological formation [10]. As small non-coding RNAs, miRNAs are responsible for regulating the expression of gene and are able to play a crucial part in inflammation, ageing, degeneration and other physiological processes [11]. Dysfunctions of miRNAs have been reported in many neurological disorders, especially in PD [12]. For instance, miR-133b can decrease α-syn expression and rescue neuronal survival [13]. On the contrary, upregulation of miR-16-1 may reduce clearance of α-syn aggregation thus contributing to loss of dopaminergic neurons [14]. Moreover, recent evidence also indicates that long ncRNAs (lncRNAs) can interact with miRNAs then involved in PD pathogenesis [14, 15]. As a focus of many scholars, lncRNA NEAT1 has been reported increasing neuronal injuries in MPP+ treated SH-SY5Y cells by combining miR-1301-3p [16]. NEAT1 could also act as a sponge of miR-212-3p therefore promoting inflammation in PD. Nevertheless, whether NEAT1 could bind to other miRNAs and the underlying mechanisms are not completely elucidated.

Based on the above, this study established PD models of mice and primary dopaminergic neurons with rich expression of α-syn, then discussed the effect of Ang-(1-7)/Mas on these models combined with downstream lncRNA and miRNA. Our work may bring new diagnostic biomarkers and therapeutic directions for PD.

## RESULTS

### Ang-(1-7) takes a protective part in the hα-syn overexpressed PD model of mice

**Figure 1:**
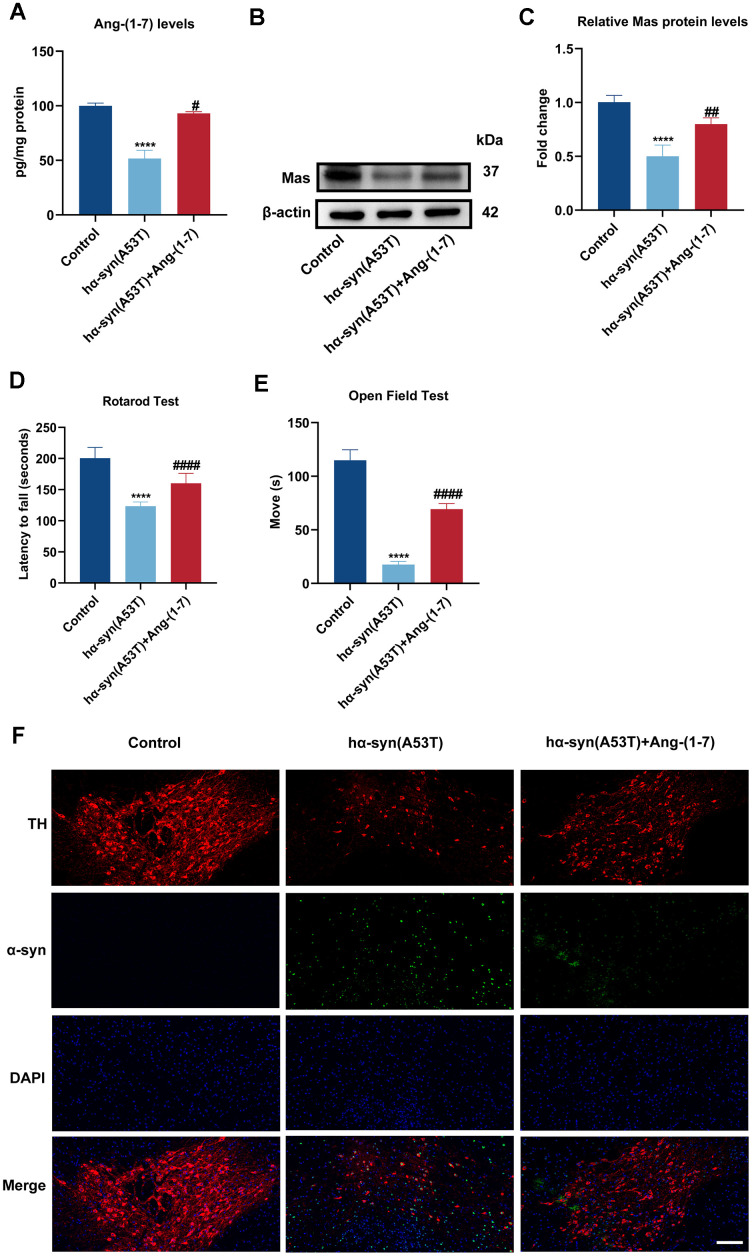
**Ang-(1-7) takes a protective part in the hα-syn overexpressed PD model of mice.** (**A**) The Ang-(1-7) levels within SN in different groups were examined using enzyme-linked immunosorbent assay (n = 6). (**B**) The MasR levels within SN in different groups were assessed by Western blot (n = 6). (**C**) Quantitative evaluation of MasR level. (**D**) The latency time in the Rotarod test were recorded (n = 21). (**E**) The movement time in the open field test were shown (n = 21). (**F**) Cells were labeled by anti-TH (red) and anti-α-syn (green) antibodies, nuclei were counterstained with DAPI (blue). The immunofluorescence was surveyed using a fluorescent microscopy. Scale bar, 100 μm (n = 6). Data are shown as the mean ± SD. ****P<0.0001 versus the Control group; #P<0.05, ##P<0.01 and ####P<0.0001 versus the hα-syn(A53T) group.

Mice received intranigral injection of AAV-hα-syn(A53T) to observe the alteration of Ang-(1-7)/MasR axis. As shown in Figure 1A–1C, the Ang-(1-7) and Mas levels in the SN of mice which injected with hα-syn(A53T) virus were both obviously reduced than that in control mice. However, the decreased levels of Ang-(1-7) and Mas were fully reversed by Ang-(1-7) infusion. Subsequently, behavior tests were performed to explore whether Ang-(1-7) exerted neuroprotective effect *in vivo*. In the Rotarod test, mice injected with hα-syn(A53T) showed obviously shorter latency time than control mice (Figure 1D). Meanwhile, in the open field test, the hα-syn(A53T) group showed significantly shorter moving time compared with the control group (Figure 1E). However, the variations were reversed by combined injection of Ang-(1-7) (Figure 1D, 1E). Finally, immunofluorescence was performed to discover the effect of Ang-(1-7) on dopaminergic neurons and α-syn expression in PD. Figure 1F showed that the amount of TH-positive neurons was markedly decreased and the expression of α-syn was distinctly increased in the hα-syn(A53T) mice. However, injection of Ang-(1-7) obviously reversed above-mentioned changes in the level of TH-positive neurons and α-syn. Above results demonstrated that Ang-(1-7) alleviated behavioral disorders, rescued dopaminergic neurons loss and lowered α-syn expression in the SN of hα-syn(A53T) mice, therefore taking a protective part in the PD model of mice.

### Overexpression of miR-153-3p reduces the level of α-syn and relieves the apoptosis in the hα-syn(A53T) overexpressed dopaminergic neurons

To identify whether miRNAs were involved in Ang-(1-7) induced neuroprotective effect in PD, we conducted high-throughput miRNA sequencing in the hα-syn(A53T) mice with or without Ang-(1-7) administration. As shown in Figure 2A, 46 differentially expressed miRNAs were chosen with |log2(fold change) |≥1 and P<s0.0001 after Ang-(1-7) injection. Among them, miR-153-3p was the only one which could bind to 3’-UTR of α-syn confirmed by targetscan software (Figure 2B). Next, the dual luciferase reported assay displayed that miR-153-3p distinctly reduced α-syn-WT luciferase activity rather than that of α-syn-Mut (Figure 2C), which also proved the bond of miR-153-3p and α-syn. Meanwhile, the miR-153-3p levels in SN of hα-syn(A53T) mice after Ang-(1-7) injection were further confirmed by RT-qPCR (Figure 2D). Lastly, we transfected miR-153-3p mimics, miR-153-3p inhibitors or their negative controls to primary dopaminergic neurons which have been treated with AAV-hα-syn(A53T) (Figure 2E). As shown in Figure 2F, 2G, compared with the NC mimic transfected group, transfection of miR-153-3p mimic significantly reduced the level of α-syn. However, transfection of miR-153-3p inhibitor obviously increased the level of α-syn compared with the NC inhibitor transfected group. At the same time, the variation of LDH leakage in each group was in line with that of α-syn (Figure 2H). Besides, we also tested the apoptosis-related proteins of neurons. As indicated by Figure 2I–2K, 2L, transfection of miR-153-3p mimic distinctly increased the level of Bcl2, whereas significantly reduced the level of Bax and cleaved caspase-3 compared with the NC mimic transfected group. However, transfection of miR-153-3p inhibitor obviously showed the opposite variation compared with the NC inhibitor transfected group. These findings reveal that α-syn is a target protein of miR-153-3p, and overexpression of miR-153-3p reduces the level of α-syn and relieves the apoptosis in the hα-syn(A53T) overexpressed dopaminergic neurons.

**Figure 2:**
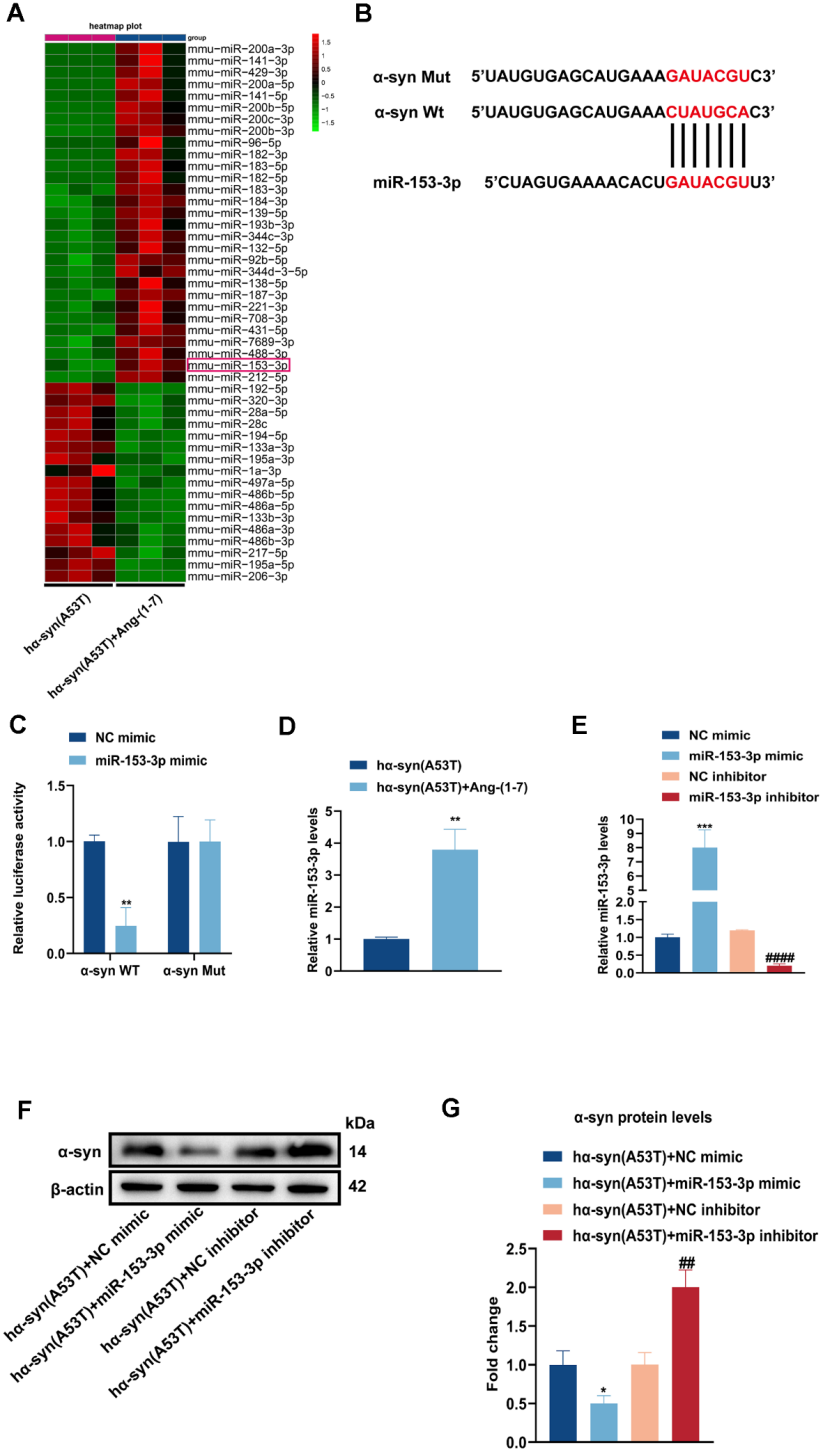
**Overexpression of miR-153-3p reduces the level of α-syn and relieves the apoptosis in the hα-syn(A53T) overexpressed dopaminergic neurons.** (**A**) The heatmap showed differentially expressed miRNAs in right SN of mice for every group (n = 3). (**B**) Probable binding site of miR-153-3p to α-syn. (**C**) The luciferase reporter assay to verify the combination between miR-153-3p and α-syn (n = 3). (**D**) The miR-153-3p level in SN of each mice group were assessed by qRT-PCR (n = 3). (**E**) The miR-153-3p levels of primary dopaminergic neurons in indicated groups were analysed by qRT-PCR (n = 3). (**F**) The α-syn level of primary dopaminergic neurons in each group was tested by Western blot (n = 3). (**G**) Quantitative evaluation of α-syn level.

**Figure 2:**
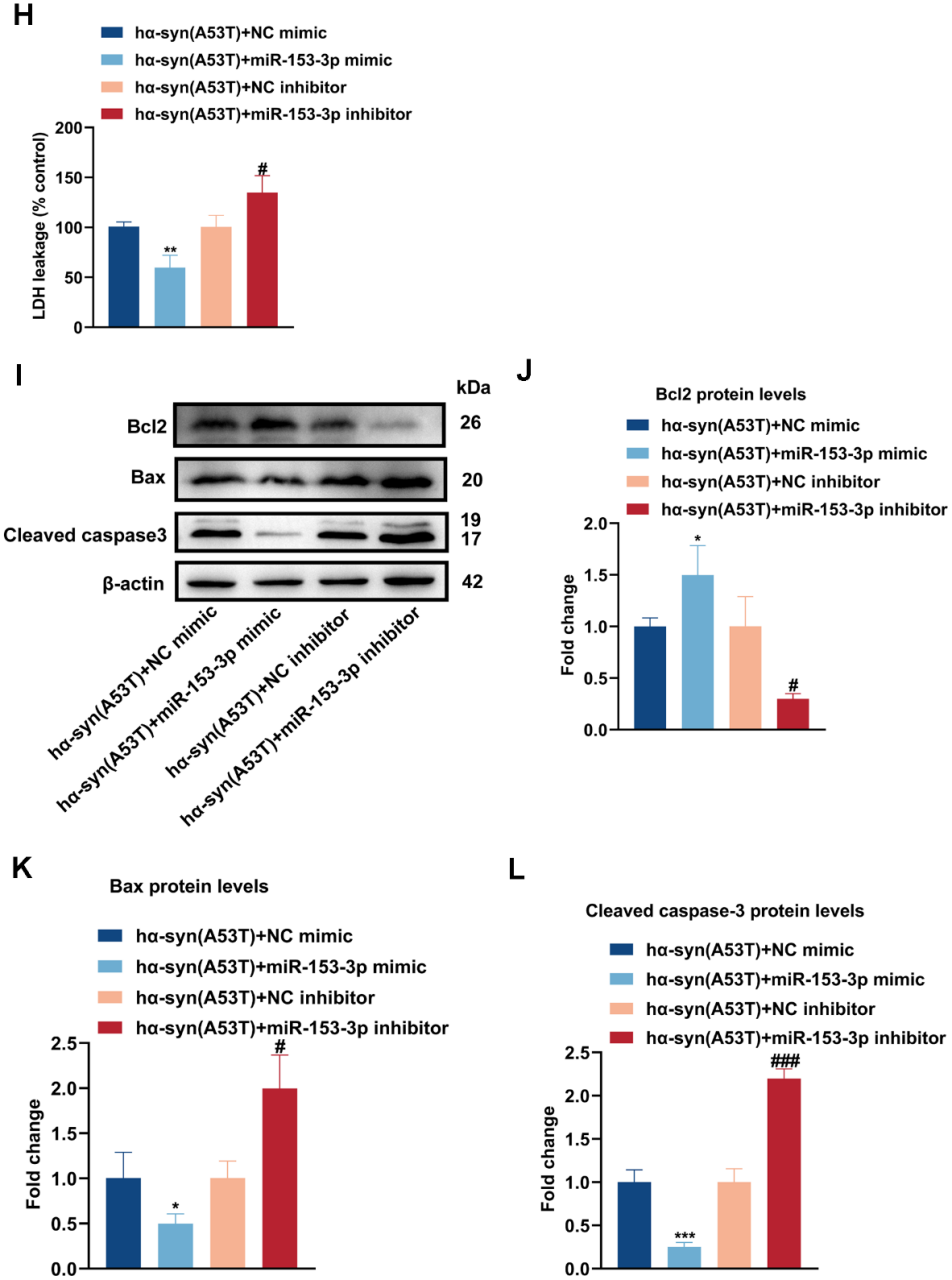
**Overexpression of miR-153-3p reduces the level of α-syn and relieves the apoptosis in the hα-syn(A53T) overexpressed dopaminergic neurons.** (**H**) Cell cytotoxicity of primary dopaminergic neurons in each group was tested by LDH assay (n = 3). (**I**) The levels of Bcl2, Bax and cleaved caspase-3 in every group were observed by Western blot (n = 3). (**J**) Quantitative evaluation of Bcl2 level. (**K**) Quantitative evaluation of Bax level. (**L**) Quantitative evaluation of Cleaved caspase-3 level. Data are shown as the mean ± SD. *P<0.05 versus the hα-syn(A53T) + NC mimic group; **P<0.01 versus the NC mimic, hα-syn(A53T) or hα-syn(A53T) + NC mimic group; ***P<0.001 versus the NC mimic or hα-syn(A53T) + NC mimic group; #P<0.05, ##P<0.01 and ###P<0.001 versus the hα-syn(A53T) + NC inhibitor group. ####P<0.0001 versus the NC inhibitor group.

### Ang-(1-7) decreases the level of α-syn and apoptosis in the hα-syn(A53T) overexpressed dopaminergic neurons through miR-153-3p

**Figure 3:**
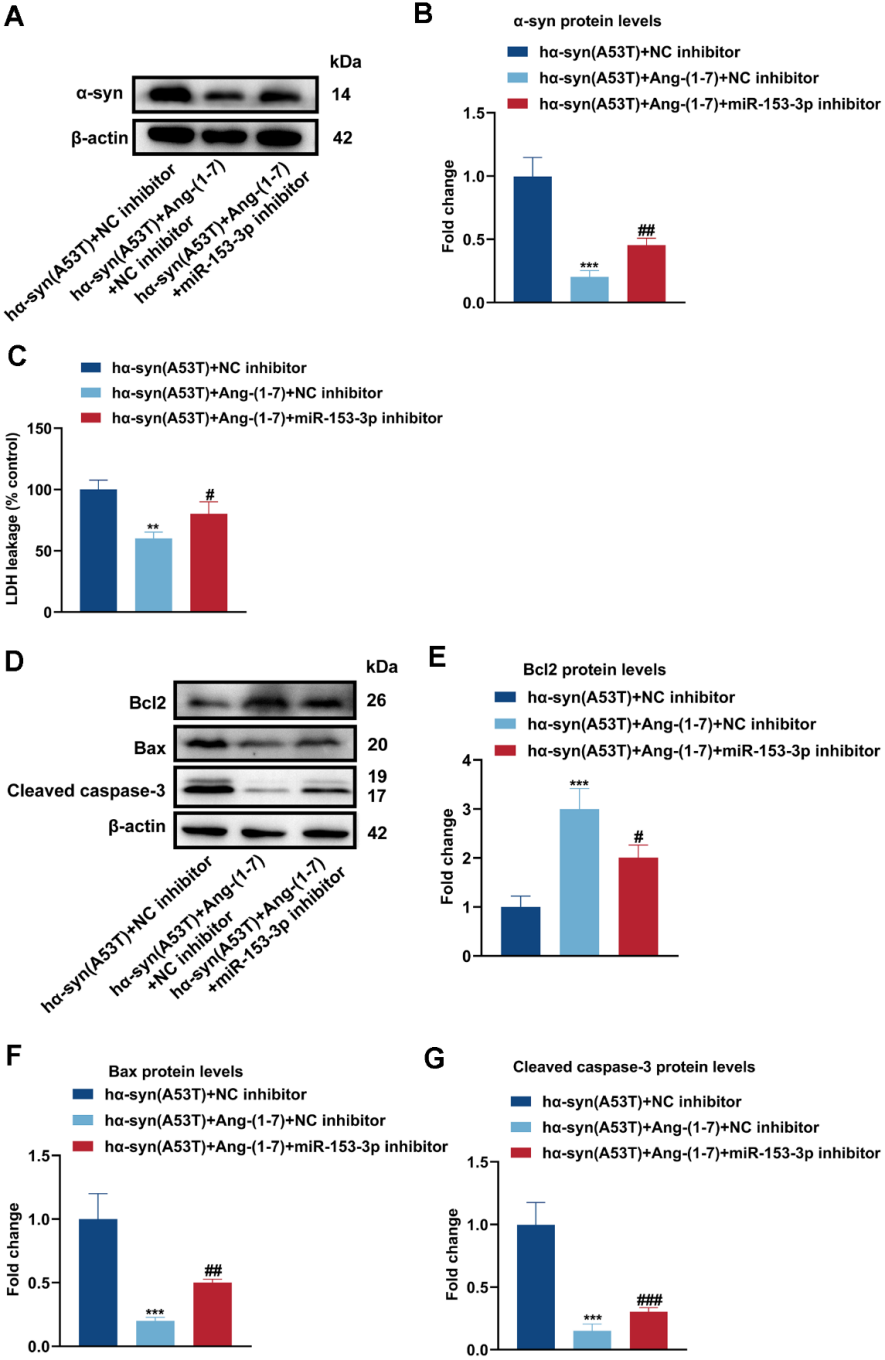
**Ang-(1-7) decreases the level of α-syn and apoptosis in the hα-syn(A53T) overexpressed dopaminergic neurons through miR-153-3p.** (**A**) The α-syn level of primary dopaminergic neurons in each group was detected by Western blot (n = 3). (**B**) Quantitative evaluation of α-syn level. (**C**) Cell cytotoxicity of primary dopaminergic neurons in each group was tested by LDH assay (n = 3). (**D**) The levels of Bcl2, Bax and cleaved caspase-3 in every group were observed by Western blot (n = 3). (**E**) Quantitative evaluation of Bcl2 level. (**F**) Quantitative evaluation of Bax level. (**G**) Quantitative evaluation of Cleaved caspase-3 level. Data are shown as the mean ± SD. **P<0.01 and ***P<0.001 versus the hα-syn(A53T) + NC inhibitor group; #P<0.05, ##P <0.01 and ###P<0.001 versus the hα-syn(A53T) + Ang-(1-7) + NC inhibitor group.

To observe whether Ang-(1-7) reduces the level of α-syn and apoptosis via miR-153-3p, Ang-(1-7), miR-153-3p inhibitor or its negative control was added to hα-syn(A53T) overexpressed dopaminergic neurons. As revealed in Figure 3A, 3B, co-treatment with Ang-(1-7) obviously inhibited the α-syn expression compared with the group treated with NC inhibitor alone. However, this variation was reversed by miR-153-3p inhibitor. Meanwhile, the LDH leakage showed the same variational tendency in all groups with that of α-syn (Figure 3C). At last, as indicated by Figure 3D–3G, Ang-(1-7) apparently enhanced the level of Bcl2 whereas reduced the level of Bax and cleaved caspase-3 compared with the NC inhibitor transfected group. However, transfection of miR-153-3p inhibitor together obviously showed the opposite variation of the three apoptosis-related proteins compared with the group treated with Ang-(1-7) and NC inhibitor. These results manifested that Ang-(1-7) decreased the level of α-syn and apoptosis in the hα-syn(A53T) overexpressed dopaminergic neurons in a miR-153-3p-dependent manner.

### NEAT1 acts as a sponge of miR-153-3p therefore increasing the level of α-syn and apoptosis in the hα-syn(A53T) overexpressed dopaminergic neurons

By searching plenty of literature and utilizing online bioinformatics databases, we found that NEAT1 had a good combination to miR-153-3p (Figure 4A, 4B). Next, dual luciferase reported assay was performed to verify the binding site. As shown in Figure 4C, miR-153-3p mimic obviously weakened the NEAT1-WT luciferase activity, rather than that of NEAT1-Mut. Then the FISH assay was conducted to define localization of NEAT1 and miR-153-3p. Figure 4D showed that NEAT1 may act as a sponge of miR-153-3p, and they were both mainly located in the cytoplasm of dopaminergic neurons. Afterwards, we knocked down or overexpressed NEAT1 in the hα-syn(A53T) overexpressed dopaminergic neurons (Figure 4E). As indicated in Figure 4F, overexpression of NEAT1 obviously lessened the miR-153-3p level whereas knockdown of NEAT1 remarkably increased the miR-153-3p level in hα-syn(A53T) overexpressed cells. Finally, we observed the influence of NEAT1 on α-syn and apoptosis in aforementioned groups. As shown in Figure 4G, 4H, overexpression of NEAT1 significantly improved the level of α-syn whereas knockdown of NEAT1 obviously decreased the level of α-syn compared to corresponding control group. At the same time, the variation of LDH leakage in each group was similar with that of α-syn (Figure 4I). Besides, we also found that NEAT1 aggravated the apoptosis. As indicated by Figure 4J–4L and 4M, overexpression of NEAT1 significantly decreased the level of Bcl2 and increased the level of Bax and cleaved caspase-3 compared to corresponding control group. However, knockdown of NEAT1 obviously showed the opposite variation of the three apoptosis-related proteins compared with the corresponding control group. To summarize, NEAT1 could bind to miR-153-3p, then increasing the level of α-syn and apoptosis in the hα-syn(A53T) overexpressed dopaminergic neurons.

**Figure 4:**
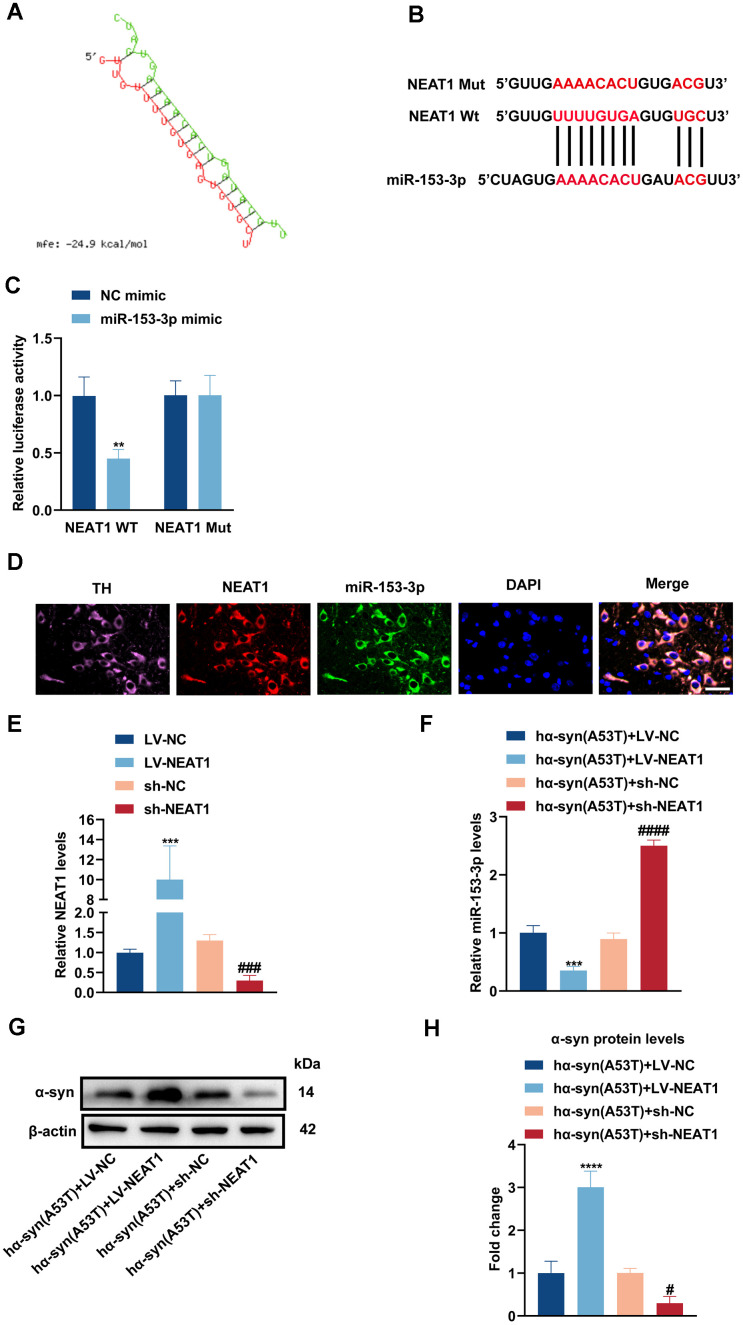
**NEAT1 acts as a sponge of miR-153-3p therefore increasing the level of α-syn and apoptosis in the hα-syn(A53T) overexpressed dopaminergic neurons.** (**A**, **B**) Probable binding site of NEAT1 to miR-153-3p. (**C**) The dual luciferase reported assay to verify the combination between NEAT1 and miR-153-3p (n = 3). (**D**) The location of NEAT1(red) and miR-153-3p(green) in the brain section of mice. TH (pink) represents the location of dopaminergic neurons, whereas DAPI (blue) represents the location of nuclear localization. Scale bar, 50 μm (n = 6). (**E**) The NEAT1 levels of primary dopaminergic neurons in indicated groups were analysed by qRT-PCR (n = 3). (**F**) The miR-153-3p levels of primary dopaminergic neurons in indicated groups were assessed by qRT-PCR (n = 3). (**G**) The α-syn level of primary dopaminergic neurons in each group was detected by Western blot (n = 3). (**H**) Quantitative evaluation of α-syn level.

**Figure 4:**
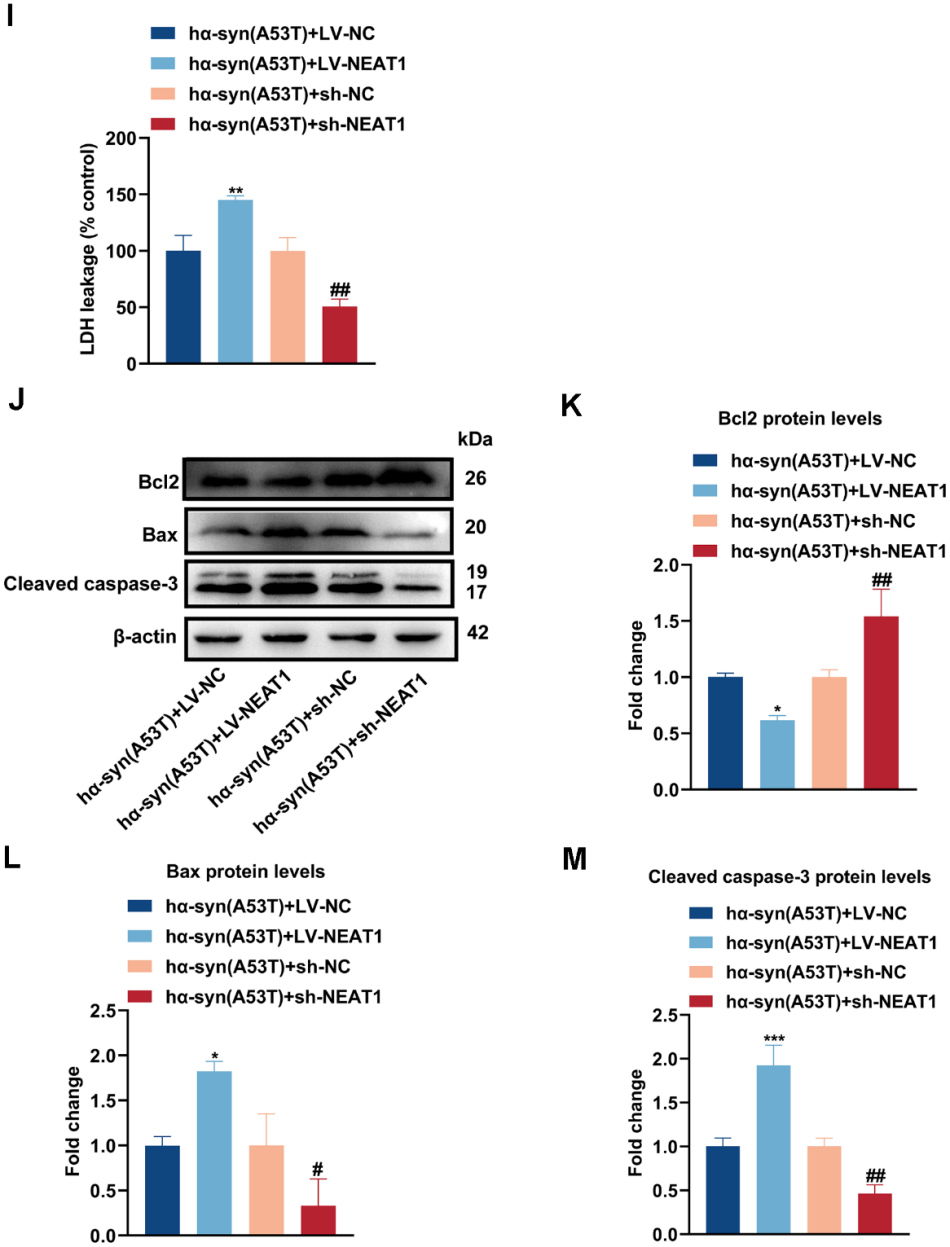
**NEAT1 acts as a sponge of miR-153-3p therefore imcreasing the level of α-syn and apoptosis in the hα-syn(A53T) overexpressed dopaminergic neurons.** (**I**) Cell cytotoxicity of primary dopaminergic neurons in each group was tested by LDH assay (n = 3). (**J**) The levels of Bcl2, Bax and cleaved caspase-3 in every group were observed by Western blot (n = 3). (**K**) Quantitative evaluation of Bcl2 level. (**L**) Quantitative evaluation of Bax level. (**M**) Quantitative evaluation of Cleaved caspase-3 level. Data are shown as the mean ± SD. *P<0.05 and ****P<0.0001 versus the hα-syn(A53T) + LV-NC group; **P<0.01 versus the NC mimic or hα-syn(A53T) + LV-NC group; ***P<0.001 versus the LV-NC or hα-syn(A53T) + LV-NC group; #P<0.05, ##P<0.01 and ####P<0.0001 versus hα-syn(A53T) + sh-NC group; ###P<0.001 versus the sh-NC group.

### Ang-(1-7) decreases the level of α-syn and apoptosis in the hα-syn(A53T) overexpressed dopaminergic neurons through NEAT1

**Figure 5:**
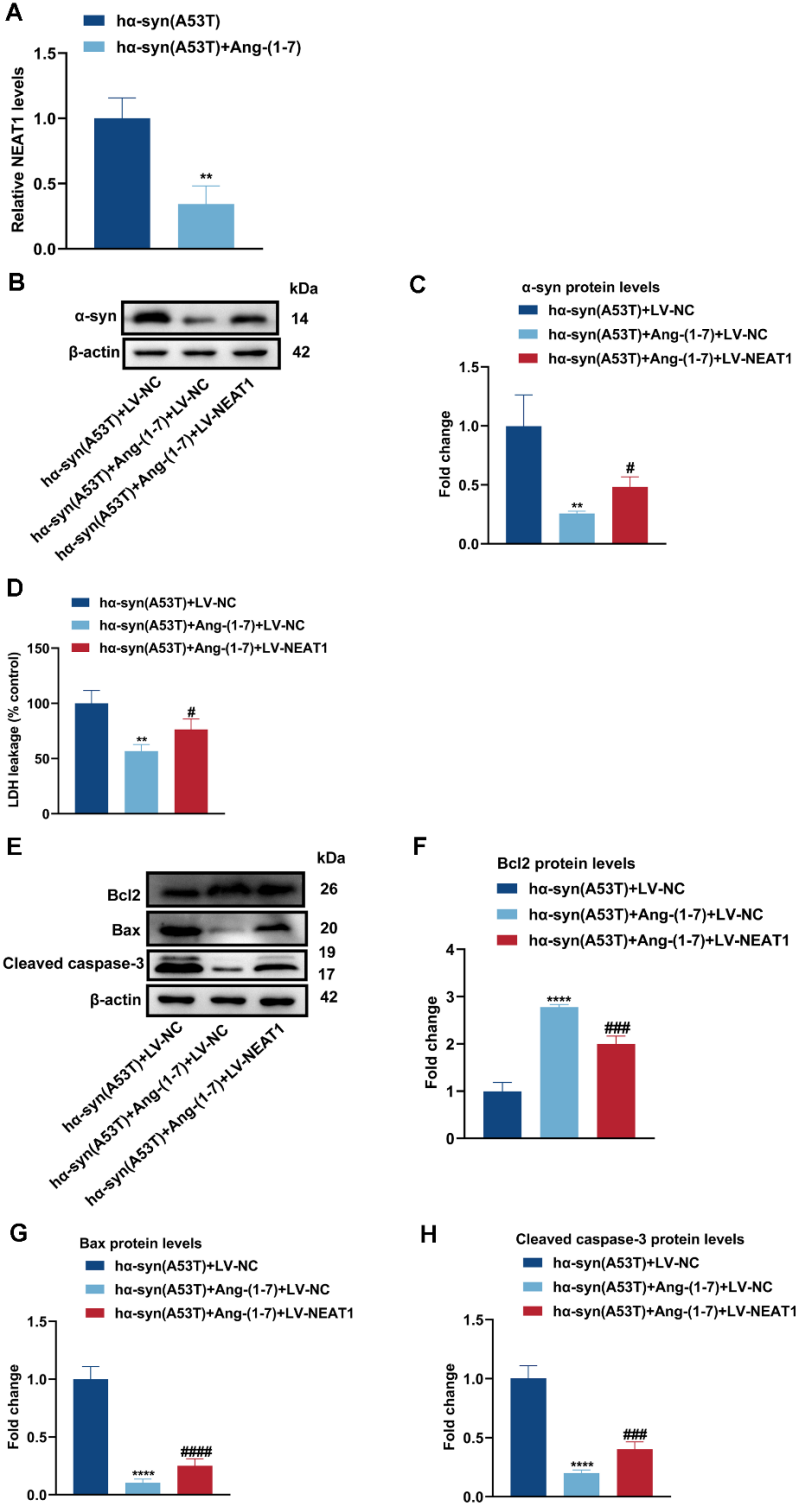
**Ang-(1-7) decreases the level of α-syn and apoptosis in the hα-syn(A53T) overexpressed dopaminergic neurons through NEAT1.** (**A**) The NEAT1 level in each group of mice was tested by qRT-PCR (n = 3). (**B**) The α-syn level of primary dopaminergic neurons in each group was shown by Western blot (n = 3). (**C**) Quantitative evaluation of α-syn level. (**D**) Cell cytotoxicity of primary dopaminergic neurons in each group was assessed by LDH assay (n = 3). (**E**) The levels of Bcl2, Bax and cleaved caspase-3 in every group were analyzed by Western blot (n = 3). (**F**) Quantitative evaluation of Bcl2 level. (**G**) Quantitative evaluation of Bax level. (**H**) Quantitative evaluation of Cleaved caspase-3 level. Data are shown as the mean ± SD. **P<0.01 versus hα-syn(A53T) or hα-syn(A53T) + LV-NC group; ****P<0.0001 versus hα-syn(A53T) + LV-NC group; #P<0.05, ###P<0.001 and ####P<0.0001 versus the hα-syn(A53T) + Ang-(1-7) + LV-NC group.

Figure 5A indicated that Ang-(1-7) obviously inhibited the expression of NEAT1 in hα-syn(A53T) overexpressed PD mice. To further explore the interaction between NEAT1 and Ang-(1-7) on the level of α-syn and apoptosis in aforementioned cells, LV-NC or LV-NEAT1 were transfected in hα-syn(A53T) overexpressed neurons. As revealed in Figure 5B, 5C, co-treatment with Ang-(1-7) apparently inhibited the level of α-syn compared with the LV-NC group. However, this variation was reversed when cells transfected with LV-NEAT1 together. Meanwhile, the LDH leakage showed the same variational tendency in three groups with that of α-syn (Figure 5D). Finally, as shown by Figure 5E–5H, Ang-(1-7) evidently heightened the level of Bcl2, whereas reduced the level of Bax and cleaved caspase-3 compared with the sh-NC transfected group. However, transfection of LV-NEAT1 together obviously showed the opposite variation of the three apoptosis-related proteins. All these conclusions showed that Ang-(1-7) decreases the level of α-syn and apoptosis in the hα-syn(A53T) overexpressed dopaminergic neurons in a NEAT1-dependent manner.

### MiR-153-3p expression in peripheral blood is negatively correlated with that of α-syn

**Figure 6:**
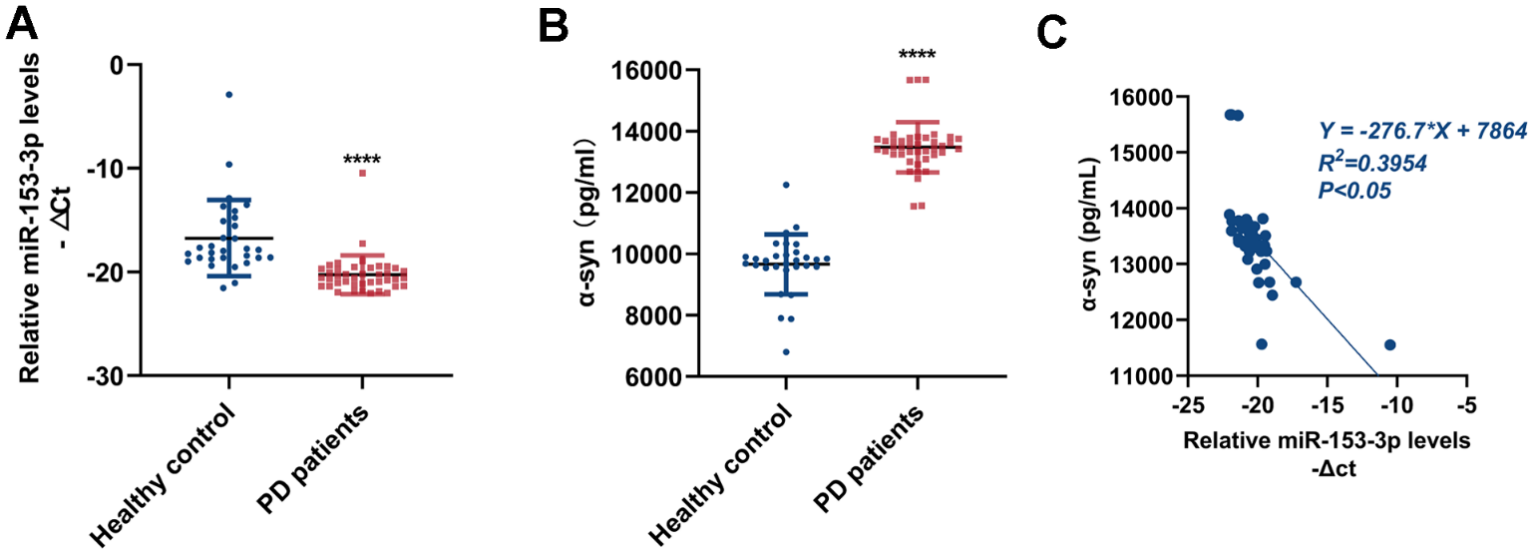
**MiR-153-3p expression in peripheral blood is negatively correlated with that of α-syn.** (**A**) The relative level of miR-153-3p within peripheral blood from healthy controls (n = 30) and PD patients (n = 41). (**B**) The expression of α-syn within peripheral blood from healthy controls (n = 30) and PD patients (n = 41). (**C**) Correlative analysis between miR-153-3p and α-syn expression within peripheral blood. Data are shown as the mean ± SD.

To explore the relationship between the expression of miR-153-3p and α-syn in peripheral blood, we took samples of blood from PD patients and healthy controls to examine miR-153-3p and α-syn expressions. As exhibited in Figure 6A, 6B, the level of miR-153-3p was obviously lower, whereas the level of α-syn was remarkably higher in the plasma of PD patients than in healthy controls. Next, we conducted the correlation analysis of the two indexes. Figure 6C suggested that the miR-153-3p level in peripheral blood is negatively correlated with that of α-syn. This finding not only clarified the correlation of miR-153-3p and α-syn, but also showed that the two indexes may be regarded as biomarkers in PD.

## DISCUSSION

Ang-(1-7)/Mas receptor axis was a recently discovered bypass of RAS, which could confront the pro-inflammatory and pro-apoptotic effects of classical Ang II/AT1 pathway [17]. Its neuroprotective role in neurodegenerative diseases, especially in PD has attracts a lot of attentions in the last few years [6, 18]. Rabie MA showed that Ang-(1-7) could exert antioxidant effects and contribute to DA synthesis increase in the PD rat model [19]. A recent study also reported that Ang-(1-7)/Mas axis had the anti-inflammatory capacity therefore hindering motor deficits in 6-hydroxydopamine hemiparkinsonian rats [20]. Here, we established a mouse model of PD which could closely simulate the pathologic feature by infecting with hα-syn(A53T) virus. The results showed that the expression of Ang-(1-7) and MasR were both obviously decreased in this PD model. Moreover, exogenous Ang-(1-7) could ameliorate the behavioral disorders, alleviate loss of dopaminergic neuron and reduce α-syn expression in PD mice. This was in line with our previous finding that Ang-(1-7) could exert anti-apoptosis effect in the rotenone-induced cell model and PD rat model [21]. All these results emphasized the conservation value of Ang-(1-7) in PD and throw a new light on the correlation between Ang-(1-7) and α-syn pathology.

MicroRNAs are small non-coding RNAs which regulate gene expression and participate in numerous physiological and pathological processes [22]. Among them, miR-153-3p has been discovered involved in several diseases including the nervous system diseases [22]. For example, it may suppress cell proliferation and invasion thus playing a role in thyroid cancer [23]. MiR-153-3p can also confer neuroprotection by attenuating oxidative stress in ischemic stroke [24]. Moreover, Zhou Q found that the miR-153-3p level was obviously lower in plasma of patients with AD, and miR-153-3p could regulate amyloid precursor protein expression thus affecting the occurrence of AD [25]. Based on these, our research used high-throughput miRNA sequencing to find differentially expressed miRNAs in PD mice with or without Ang-(1-7) injection. MiR-153-3p was selected to be the only miRNA which showed distinctly differential expression and could bind to 3’-UTR of α-syn. In addition, we detected that miR-153-3p could lower the α-syn expression and reduce the apoptosis in the hα-syn(A53T) overexpressed dopaminergic neurons. These findings were not only consistent with previous conclusions that miRNAs may be regarded as potential biomarkers for PD [26, 27], but also offer a potential site in treating this troublesome disease.

It is well known that lncRNAs could influence the level of miRNA and then take part in many pathological processes including neurodegenerative disorders [15, 28]. For instance, lncRNA MIAT downregulation could increase miR-150-5p levels, thus reducing Aβ clearance and playing a crucial role in mouse model of AD [29]. Meanwhile, upregulation of lncRNA NEAT1 contributes to increasing miR-107 activity, therefore aggravating Aβ expression and pTau in AD models [30]. In addition to AD, NEAT1 has also drawn lots of attentions to its relation with PD [31]. Xie SP et al. have discovered that NEAT1 could target miR-124 and increase neuronal injury in MPTP-treated SH-SY5Y cells [32]. Our present study showed that NEAT1 could not only bind to miR-153-3p, but also aggravate the level of α-syn and apoptosis in the hα-syn(A53T) overexpressed dopaminergic neurons. This discovery was supported by several studies indicating that NEAT1 played an important part in PD via acting as miRNA sponge [16, 33]. Besides, we also found that Ang-(1-7) could decrease the α-syn and apoptosis expressions in the cell model through downregulating NEAT1. As far as we know, this research is the first to show that Ang-(1-7) can regulate the progress of PD by targeting NEAT1.

A growing number of evidence suggested that miRNA expressions may help diagnosis and prognosis for PD patients [34]. For example, both pre-clinical and clinical clues have identified miR-124 level may act as a promising diagnostic biomarker in PD [35]. Moreover, miRNA-based analysis combined with α-syn data could reflect probable progress [36]. Dos Santos et al. have found a miRNA-based biomarker panel from patients’ CSF for early diagnosis of PD, meanwhile, join of α-syn in the analysis enhances robustness of the panel [37]. Similarly, our research revealed that miR-153-3p expression from patients’ blood sample is negatively correlated with that of α-syn. This brings more hope for clinical application of miRNA detection.

However, there are some limitations in our study. First of all, we didn’t explain how Ang-(1-7) regulates NEAT1. Actually, N6-methyladenosine(m6A) sites have been identified on NEAT1 [38], thus the m6A level can affect the expression of NEAT1. Based on this, we suspect that Ang-(1-7) may regulate NEAT1 through influencing its m6A modification level. In addition, the related mechanisms between Ang-(1-7) and PD were verified in the cell model of PD, but it has not been validated *in vivo*. Relevant exploration will be carried out in the follow-up experiments.

Taken together, we demonstrated for the first time that by targeting NEAT1/miR-153-3p axis, Ang-(1-7) can lower the α-syn and apoptosis level, and relieve the behavioral disorder in hα-syn(A53T) overexpressed PD models. This not only uncovered the significance and related mechanisms of Ang-(1-7) on α-syn development, but also threw a new light upon miR-153-3p and NEAT1 as biomarkers and therapeutic targets in PD.

## MATERIALS AND METHODS

### Animals and treatments

Male C57BL/6J mice weighing 20-25 g were purchased from Vital River Laboratories (Beijing). The mice were kept in an air-conditioned space with temperature (22 ± 2° C), humidity (50–60%) and with enough food and water. The animal studies were conducted under the permission of the Animal Care and Use Committee of Nanjing First Hospital (Number: DWSY-22003128).

Mice were randomly arranged for three groups: Control group and hα-syn(A53T) group received supranigral injection of artificial cerebrospinal fluid (aCSF) (0.25 μL/hour) for 4 weeks, hα-syn(A53T) + Ang-(1-7) group received supranigral injection of Ang-(1-7) (1.1 nmol/0.25μL/hour, Sigma-Aldrich, St. Louis, MO, USA) for 4 weeks. The procedure and dosage for this experiment is following our previous study [39].

### Stereotaxic injection

As previously described [40], after anesthetized with isoflurane in 2% oxygen-enriched air, the 8-week-old mice received injection of AAV-hα-syn(A53T) (Genechem Co., Ltd, Shanghai, China) within the right SNpc (Anteroposterior: -2.9mm; Mediolateral: -1.3mm; Dorsoventral: -4.5 mm from bregma) to establish the PD model. The aCSF or Ang-(1-7) was injected in the same location.

### Assessment of Ang-(1-7) level

Briefly, the SN portions were isolated following the 4-week injection of vehicle or Ang-(1–7), then homogenized and centrifuged to get rid of cellular debris. The supernatant was conserved for use. According to previous description [21], the level of Ang-(1–7) within the SN was examined with enzyme-linked immunosorbent assay (S-1330, BMA Biomedicals, Augst, Switzerland).

### Behavioral tests

### Rotarod test

The rotarod test was conducted to assess motor coordination using a rotarod instrument [41]. Mice were put on the instrument with rotating speed from initial 5 rpm accelerating to 40 rpm in 5 min. The maintaining time for the mouse staying on the rotarod was recorded as the latency time.

### Open field test

The open field test was operated in a square place (47 cm× 47 cm × 40 cm) for 5 min to assess spontaneous motion of mice [42]. The moving time of the mouse was recorded by TSE system (TSE Systems, Bad Homburg, Hesse-Darmstadt, Germany).

### Immunofluorescence analysis

Immunofluorescence analysis was performed as described [21]. The right SN parts from aforementioned groups were fixed for 10 min in 4% paraformaldehyde and cut into 5-μm-thick parts. After dewaxed, hydrated, and soaked in H_2_O_2_, sections were dealt with 0.5% Triton X-100, blocked by 5% bovine serum albumin, and then cultured at 4° C with primary antibodies overnight: TH (1:1000, AB152, Sigma-Aldrich, St. Louis, MO, USA) and α-syn (1:100, MA5-45837, Thermo Fisher Scientific, Waltham, MA, USA). The next day, samples were rinsed by PBS, cultured with conjugated secondary antibodies Alexa Fluor 594 (red) (1:200, ab150084, Abcam, Cambridge, MA, USA) and Alexa Fluor 488 (green) (1:200, ab150113, Abcam) for 1 h at 37° C. The nuclei were counterstained with 4′,6-diamidino-2-phenylindole (DAPI) (Vector Laboratories, Burlingame, CA, USA) and finally surveyed using a fluorescent microscopy (Olympus Corporation, Tokyo, Japan).

### miRNA sequencing

RNA was isolated from the right SN of mice using Trizol (Thermo Fisher Scientific, Waltham, MA, USA) referring to the directions [43]. RNA library was prepared with 1 μg of DNase-treated total RNA as input, and quantified with ABI 9700HT Fast Real-Time PCR system. Sequencing was conducted with NovaSeq platform (Illumina) which producing 150bp paired-end reads. Bowtie-v1.2.3 was used to match clean reads to the reference genome. DESeq2-v1.10.1 was used to normalize the mapped reads and analyze differential expression of miRNA. |log2(fold change) |≥1 was regarded as differentially expressed miRNAs and P<0.0001 was considered significant. The sequencing result was saved in GenBank (GSE248268).

### Dual luciferase reported assay

We used TargetScan 7.2 prediction software (http://www.targetscan.org/) and RNAhybrid (https://bibiserv.cebitec.uni-bielefeld.de/rnahybrid/) bioinformatics software to predict the probable sites of α-syn 3’-UTR binding to miR-153-3p and feasible sites of miR-153-3p binding to NEAT1. The NEAT1 sequences and its mutant were constructed. The Mut or WT of α-syn 3ʹ-UTR or NEAT1 luciferase reporter products were transfected into HEK 293T cells with NC mimic or miR-153-3p mimic (GenePharma, Shanghai, China) by lipofectamine 2000 (Thermo Fisher Scientific, Waltham, MA, USA). Cells were surveyed using a dual luciferase reporting analysis system (Promega, Madiso, WI, USA) after treatment for 48h.

### qRT-PCR

Total RNA was extracted from tissues or cells with Trizol (Thermo Fisher Scientific, Waltham, MA, USA) and then reversely transcribed with a cDNA Synthesis Supermix (TAKARA, Kusatsu, Shiga, Japan) after assessing the concentration. The cDNA was examined by the SYBR qPCR Supermix Plus (TAKARA, Kusatsu, Shiga, Japan). β-actin and U6 were respectively regarded as internal controls for mRNA and miRNA. The primers of qRT-PCR were listed in Supplementary Table 1.

### Cell culture and treatment

Primary dopaminergic neurons from the midbrain of mouse were collected following a previous study [44]. The midbrain was separated of gestational day 14 mouse embryos, digested, filtered, and then centrifuged for 5 min. Next, the cells were transferred to DMEM medium with 10% fetal bovine serum and cultured in plates containing poly-L-lysine with 5% CO_2_ for 4 hours at 37° C. Finally, the medium was turned to Neurobasal medium with 2% B27 and was replaced every 2 days. It cost 8 days for maturation to the dopaminergic neurons.

MiR-153-3p mimic, miR-153-3p inhibitor and their negative controls (NC mimic, NC inhibitor) were obtained from Shanghai GenePharma Company. hα-syn(A53T), Lenti-NEAT1, sh-NEAT1 and respective lentiviral scramble controls were obtained from Shanghai GeneChem Company. According to the instruction, above vectors were transfected with Lipofectamine 2000 to cells. Puromycin was co-cultured in Lentivirus infected cells to remain stable. The transfection efficiency was tested by qPCR or western blot.

### Western blot

Proteins from mouse midbrain or primary dopaminergic neurons were collected by RIPA lysate (Beyotime, Shanghai, China). The same amount protein samples of all groups were separated using sodium dodecyl sulfate-polyacrylamide gels electrophoresis and transferred to the membranes. After blocked in 5% non-fat milk, the membranes were treated with primary antibodies at 4° C overnight: anti-MAS1 (1:1000; #PA5-97953; Thermo Fisher Scientific, Waltham, MA, USA), anti-α-syn (1:1000; #PA5-85791, Thermo Fisher Scientific, Waltham, MA, USA), anti-Bcl2 (1:1000; ab196495; Abcam, Cambridge, MA, USA), anti-Bax (1:1000; #2772, Cell Signaling Technology, Beverly, MA, USA), anti-Cleaved caspase-3 (1:1000; #9661; Cell Signaling Technology, Beverly, MA, USA) and β-actin (1:1000; #4970; Cell Signaling Technology, Beverly, MA, USA). All membranes were rinsed for thrice and cultured by HRP labeled secondary antibody (1:3000; #7074; Cell Signaling Technology, Beverly, MA, USA). At last, the protein bands were observed by an ECL kit (Thermo Fisher Scientific) and gray value analysed by ImageJ software (NIH, Bethesda, MD, USA).

### Lactate dehydrogenase (LDH) cytotoxicity assay

The LDH cytotoxicity assay was used to assess the cytotoxicity in different treated groups. In brief, cells were evaluated using a LDH-Cytotoxicity Assay Kit (Abcam, Cambridge, MA, USA) on the basis of a previous study [45], and the absorbance of formazan was recorded with a microplate reader (Thermo Fisher Scientific, Waltham, MA, USA) at the wavelength of 500 nm.

### Fluorescent *in situ* hybridization (FISH)

MiR-153-3p and NEAT1 probes were obtained from RiboBio Co., Ltd (Guangzhou, China). According to the instruction, frozen slices of mice brains were performed *in situ* hybridization with the two probes, blocked and then cultured at 4° C with anti-TH (1:100; #45648, Cell Signaling Technology, Beverly, MA, USA). The next day, slices were rinsed for thrice and treated by Cy5-labeled goat anti-mouse IgG (1:200; GB27301, Servicebio, Wuhan, China). DAPI was applied for staining the nuclei and slices were finally observed by a confocal microscope (Zeiss Laboratories, Thornwood, NY, USA).

### Clinical data

PD patients and healthy controls (healthy individuals with no neurological diseases or family history of PD) from 2021 to 2022 admitted to Nanjing First Hospital took part in this study and have written informed consent. The experiment was allowed by the ethical committee of Nanjing First Hospital (Number: KY20190509-05-KS-01). The PD patients got diagnosis according to the International Parkinson and Movement Disorder Society clinical diagnostic criteria for PD [46]. Exclusion criteria included (1) atypical or secondary parkinsonism; (2) other neurologic injury (traumatic brain injury, stroke, Alzheimer’s disease, epilepsy); (3) acute or chronic inflammatory diseases; (4) cardiovascular disease or malignancies; (5) unstable psychiatric disorders such as schizophrenia or major depression. Healthy controls had no neurological diseases or family history of PD. There was no difference in the age and gender between the two groups. Blood samples from the participants were centrifugated and the upper plasma layer were collected to detect the α-syn expression by enzyme-linked immunosorbent assay (ELISA) kit (KHB0061, Thermo Fisher Scientific, Waltham, MA, USA). The lower samples were separated for RNA and then used to measure the miR-153-3p level by qRT-PCR.

### Statistical analysis

Statistical analyses were operated by the Prism 8.0 software (GraphPad, La Jolla, CA, USA). Data were shown as mean ± SD. Differences between two or multiple groups were respectively examined by Student’s t-test and one-way ANOVA. Correlations between the level of α-syn and miR-153-3p in peripheral blood were calculated by the Pearson’s method. P <0.05 was regarded as statistical significance.

## Supplementary Material

Supplementary Table 1
