# Supplementary material for: Angiotensin-(1-7) relieves behavioral defects and α-synuclein expression through NEAT1/miR-153-3p axis in Parkinson’s disease
Source: Aging (Albany NY). 2024 Oct 17;16(21):13304–22. doi: 10.18632/aging.206028 (PMC11719108; doi:10.18632/aging.206028)
Supplement: Supplementary Table 1 [file aging-16-206028-s001.pdf]

SUPPLEMENTARY TABLE

Supplementary Table 1. Primer sequences for qRT-PCR.

Name	Sequence
mmu-NEAT1	F: 5’- TTTGCCTAGGTTCCGTGCTT-3′<br>R: 5’- CATCCTCCACAGGCTTACCG -3′
miR-153-3p	F: 5’- TTGCATAGTCACAAAAGTGATC -3′<br>R:5’- GATCACTTTTGTGACTATGCAA -3′
β-actin	F: 5’- CCACCATGTACCCAGGCATT-3′<br>R: 5’- CGGACTCATCGTACTCCTGC-3′
U6	F: 5’-AGAGAAGATTAGCATGGCCCCTGC-3′<br>R: 5’-ATCCAGTGCAGGGTCCGAGG-3′
